# Functional results of hautmann neobladder with chimney modification and wallace ureteroileal anastomosis: initial experience with 22 patients

**DOI:** 10.1590/S1677-5538.IBJU.2020.0415

**Published:** 2021-02-03

**Authors:** Dejan Djordjevic, Marko Vukovic

**Affiliations:** Clinical Centre of Serbia Clinic Urology Department of Urology Belgrade Serbia Department of Urology, Clinic Urology, Clinical Centre of Serbia, Belgrade, Serbia; Clinical Centre of Montenegro Clinic Urology Department of Urology Podgorica Montenegro Department of Urology, Clinic Urology, Clinical Centre of Montenegro, Podgorica, Montenegro

**Keywords:** Urinary Bladder Neoplasms, Diagnostic Techniques, Surgical, Reconstructive Surgical Procedures

## Abstract

**Objective:**

To assess the functional outcomes and complications of modified Hautmann neobladder with Wallace ureteroileal anastomosis on a 6-8 cm long isoperistaltic chimney, following radical cystectomy.

**Materials and Methods:**

Between January 2015 and October 2019, 22 patients (18 men and 4 women) underwent radical cystectomy and Hautmann neobladder reconstruction with chimney modification and Wallace I ureteroileal anastomosis. The mean age of patients was 61 years (45–74 years). All procedures were performed by the same surgeon and the mean follow-up was 29.4 months. Complications were registered as early (occurring within 3 months) or late (occurring after 3 months), with particular attention addressed to the ureteroileal anastomotic stricture and anastomotic leakage rate. Patient evaluation also included symptom analysis for daytime continence and voiding frequency.

**Results:**

Ureteroileal anastomotic stricture was not detected as a cause of hydronephrosis. Hovewer, the anastomotic leakage occurred in one patient during the early postoperative period. Early complications occurred in 9 patients and the most common was bilateral hydronephrosis, detected in 5 examinees. Late complications occurred in 4 patients. Complete daytime and nighttime continence achieved in 18 and 16 patients respectively, with two patients (9%) still required intermittent catheterization three months after surgery.

**Conclusions:**

The functional results with modified Hautmann neobladder, incorporating short afferent limb in Wallace I uretero-enteric anastomosis, were efficient. This technique is an effective way to minimize potential uretero-enteric stricture, anastomotic leakage and incidence of vesicoureteral reflux.

## INTRODUCTION

Orthotopic ileal neobladder is one of the most common techniques currently employed for bladder replacement. The modification of the Hautmann ileal neobladder has already been described (1, 2), and compares favorably to other forms of urinary diversion with regards to ureteral stenosis, urinary incontinence and simplification of the uretero-intestinal anastomosis. Under cases of recurrent disease, it seems to provide greater versatility for short ureters and easy post-operative access for uretero-intestinal anastomotic revision or resection. The standard technique for uretero-enteric anastomosis is Bricker ureteral implantation in an end-to-side fashion using running sutures (3), where benign uretero-ileal anastomotic stricture (UIS) was reported with an incidence ranging from 2.7% to 10%. Additionally, there is a significant risk of vesicoureteral reflux (VUR) occurrence and subsequent upper urinary tract infection. These drawbacks could be partially corrected by modified technique of the Hautmann neobladder using a single, short chimney, in order to allow the use of longer segments of the lower ureters which participate in reflux prevention. Moreover, some authors emphasize the advantage of Wallace anastomosis over the Bricker technique. It seems that conjoined ureteral plate and larger diameter for uretero-ileal anastomosis may be associated with less tension on the ureteral walls, which ultimately results in absence of UIS (3, 4).

The objective of this study was to describe our technique of Hautmann neobladder with chimney modification and Wallace I uretero-ileal anastomosis and to compare our results with present literature in relation to peri-operative functional outcomes and complications.

## MATERIALS AND METHODS

### Patients and study design

Between January 2015 and February 2019, an orthotopic neobladder reconstruction was performed in 22 patients who had organ confined bladder cancer and who were scheduled for radical cystectomy (RC). All surgical procedures were performed by a single expert and high-volume surgeon (D. Dj), who has ample experience (>10 years) in radical cystectomy with Hautmann ileal neobladder prior to this study. Eligible patients were aged ≥40 years (yr) and had bladder cancer clinical stage T2-T3/N0-3/M0. Patients were excluded if they had previous pelvic radiation, history of inflammatory bowel disease or small bowel surgery, clinical stage T4 or M1, or prior extensive abdominal surgery. Males with prostatic urethral involvement were also excluded from the study as well as females with tumor extension to the bladder neck region or anterior vaginal wall. Since only a small bowel was used, no specific bowel preparation was necessary. An oral fluid diet was given one day before surgery, which was stopped six hours before anesthetic induction. Post-surgery, each patient was re-evaluated on a 3-month basis for one year, 6-monthly for the second year and annually thereafter. Complications were reported according to the modified Clavien-Dindo classification system (5). Reservoir-related complications included obstructive or non-obstructive hydronephrosis, UIS, pyelonephritis, anastomotic leakage, metabolic acidosis and VUR. The surgical protocol had been approved by the University of Belgrade institutional review board and registered, in view of the retrospective nature of the study, with the Ethical Committee of Clinical Centre of Serbia and conducted in accordance with the principles of the Declaration of Helsinki of World Medical Association. All patients provided informed consent for surgery.

### Surgical technique

**Figure 1:**
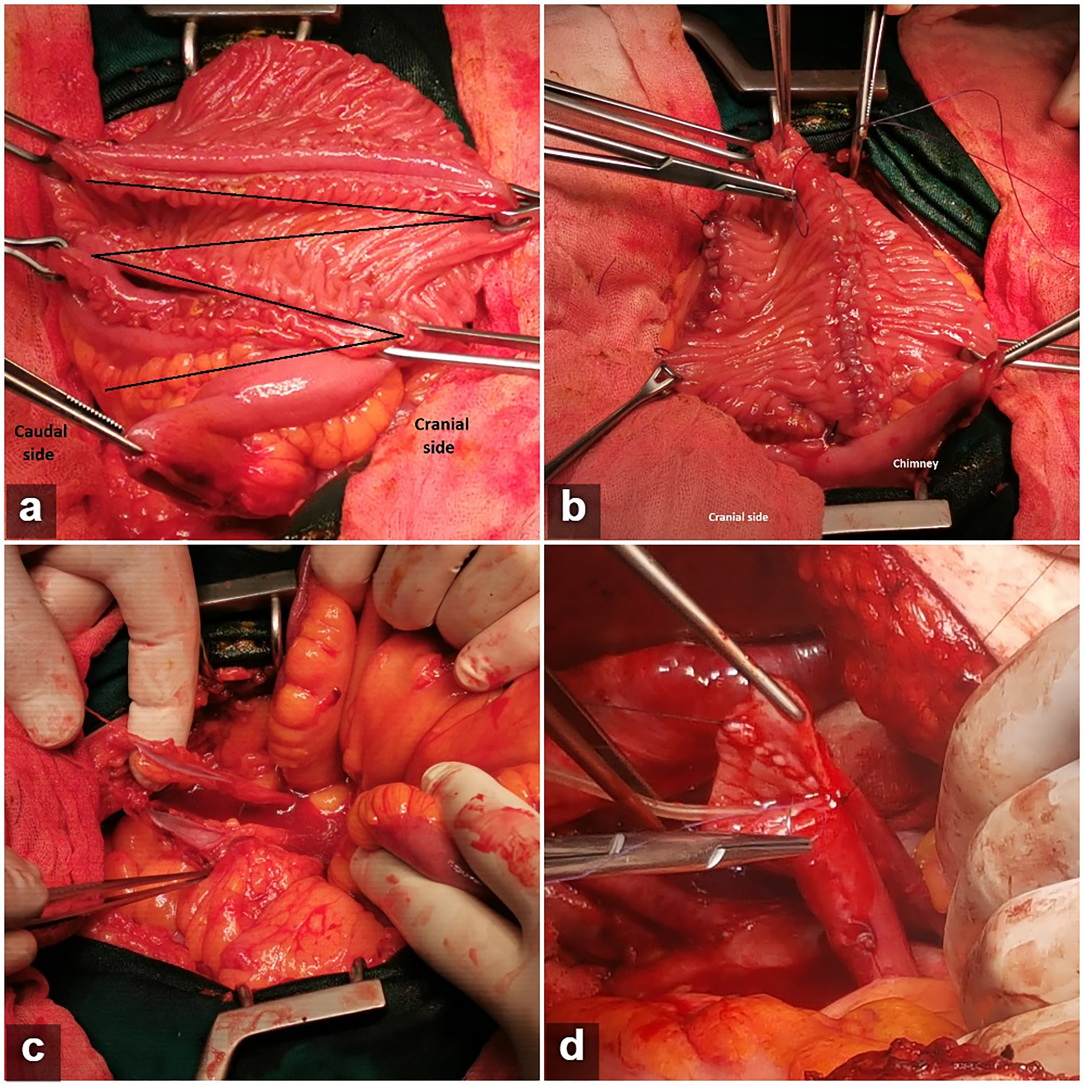
Construction of the reservoir: a) four folds of ileum were arranged in the shape of a ‘W’ using four traction points, with a 6-8cm long chimney situated on the left side of the ‘W’. Base of the “W” is oriented cranially; b) Folding of the posterior plate: an ileal plate is formed from the ‘W’ by sewing together the cut edges of the antimesenteric borders using absorbable running suture on a straight needle. Chimney is positioned on the left side of the plate; c) Ureters were spatulated for an average of 3-4cm, with maximum length of a healthy ureter and periureteral fatty tissue preserved on both sides; d) conjoined ureters sec. Wallace I.

**Figure 2:**
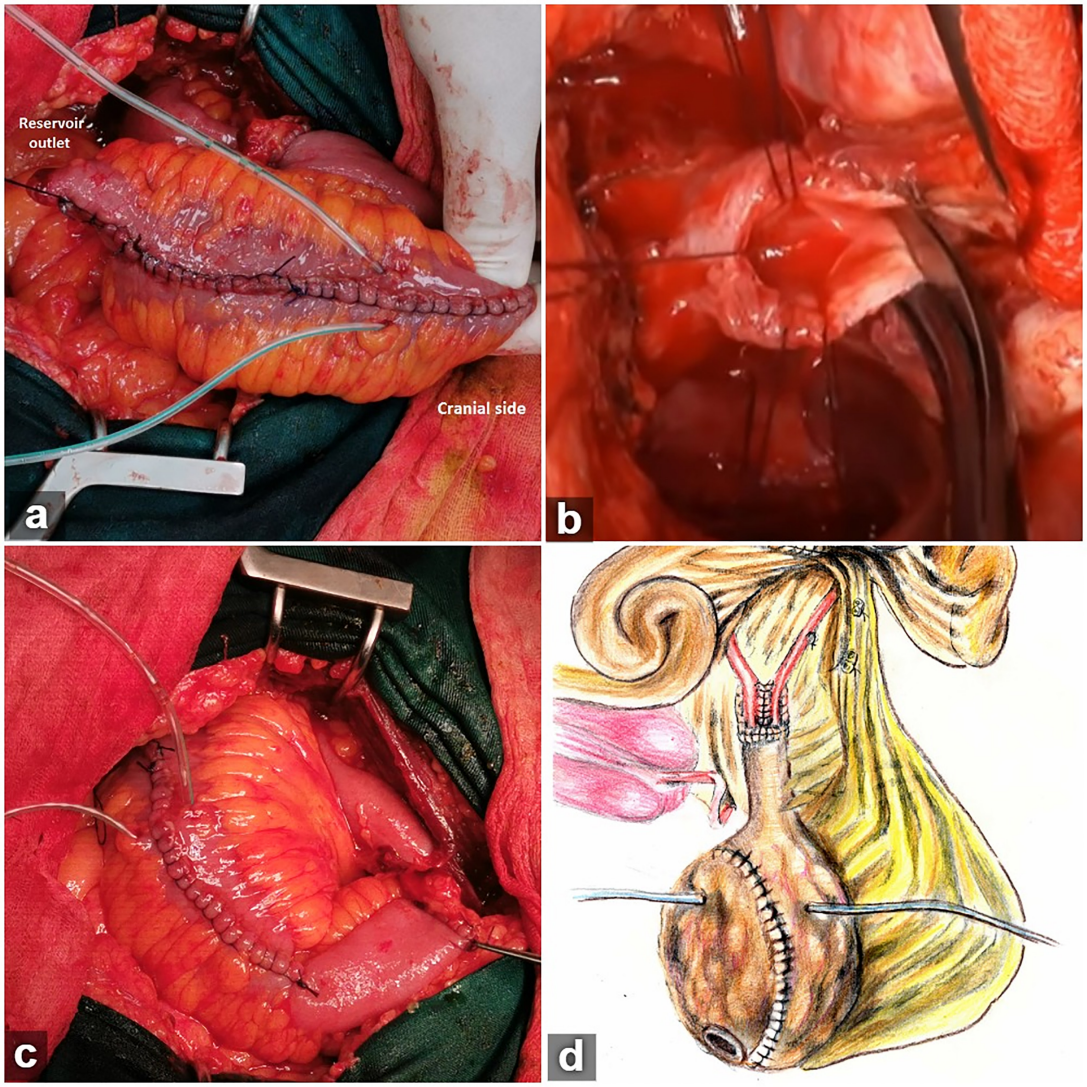
a) After closing the lower half of the anterior wall, the outlet was created as a buttonhole at the most dependent part of the pouch. After appropriate ureteral stents were placed and brought through the anterior neobladder wall, the remaining pouch wall was closed, leaving the reservoir outlet open; b) The urethral remnant has been approached and prepared exactly as is done in nerve sparing radical prostatectomy. For the ileo-urethral anastomosis, six double-armed sutures were placed, beginning with the most ventral sutures at the 1 and 11 o’clock position, followed by the sutures at the 3 and 9 o’clock position, and finally the two most dorsal sutures at the 5 and 7 o’clock position. c) final reservoir shape with ureteral stents placed through the anterior neobladder wall. No cystostomy tube was placed; d) illustration depicts main aspects of our technique and differences in comparison with the original approach.

The surgery comprised RC with standard pelvic lymph node dissection, which was followed by reconstruction of Hautmann ileal neobladder with chimney modification as follows. For constructing the reservoir, an ileal segment of approximately 55-60cm was isolated, 25cm proximal to the ileo-cecal valve, and bowel continuity restored with a 4-0 PDS single-layer seromuscular running suture. The most proximal part of the isolated ileal segment (6-8cm) was not detubularized. The remaining distal end of the ileal segment was opened along its antimesenteric border and four folds of the ileum were arranged in the shape of a ‘W’ using four traction points, with a 6-8cm long isoperistaltic chimney on the left side of the ‘W’ (Figure-1a). An ileal plate is formed by sewing together the cut edges of the antimesenteric borders using 3-0 absorbable suture (Figure-1b), meanwhile, ureters were spatulated over 3-4cm and cannulated with 6Ch ureteral catheters (Figure-1c), the maximum length of a healthy ureter was preserved on both sides while the left ureter was mobilized retroperitoneally to the right side of the pelvis, through a tunnel prepared at the base of the sigmoid mesentery. Next, ureters were anastomosed together in a running fashion, starting at the lowest point of spatulations (Figure-1d). Furthermore, free edges of the newly constructed ureteral plate were anastomosed to the open end of the chimney in an end-to-end fashion (Wallace I technique), using two running 4-0 polydioxanone sutures, starting at the lowest point of the ureteral plate and finishing at the opposite end of the newly constructed uretero-ileal anastomosis. All layers in the ureter were sutured to all layers in the ileum (6). After appropriate ureteral stents were placed, they were brought through the anterior neobladder suture line and the remaining anterior neobladder wall was closed by sewing the outer walls together using running a 3-0 synthetic absorbable suture, starting at the most caudal portion where a button-hole of all layers is excised from the ileal plate, 1-2cm from the tip of the U-shaped flap (Figure-2a). Preparation of the urethra (Figure-2b) and ileo-urethral anastomosis were performed using the same principles described in the original Hautmann technique, with a 20-22F catheter placed through the button-hole of the ileal plate (3). No cystostomy tube was placed (Figure-2c).

As clearly outlined above, the main differences in our technique from the original depiction of modified chimney technique (1, 2) were the chimney size, length of the ureteral spatulation and the end-to-end running suture uretero-ileal anastomosis (Figure-2d).

### Outcome measures and follow-up

After surgery, the transurethral catheter was required to be flushed using sterile saline every 4-6 hours (hr). Patients were told to empty neobladder initially every 2 hr in a seated position, with voiding intervals extended to 4-6 hr, which would gradually extend neobladder capacity and provide socially acceptable voiding interval. The acidosis was monitored using the base excess that was estimated by venous blood gas analysis, initially every three days, followed by weekly, depending on the blood gas values. Oral sodium bicarbonate substitution was administered for two months postoperatively to decrease the incidence of metabolic acidosis (7).

Routine follow-up examinations included laboratory studies, urinary cytology, abdominal ultrasound and intravenous urography. Abdominal/pelvic computer tomography and chest radiography were performed annually or in suspected cases of local or distal tumor progression. Patient evaluation included symptom analysis for daytime continence, enuresis and voiding frequency. They were all examined for UIS, VUR, hydronephrosis and other peri-operative outcomes during the follow-up period. Non-obstructive hydronephrosis was defined as a dilated intrarenal collecting system on imaging without evidence of UIS or other mechanical obstruction and was confirmed by intravenous urography or computer tomography. The European Organization for the Research and Treatment of Cancer Quality-of-Life Core Questionnaire version three was used to measure health-related quality of life (HRQoL). Questionnaires were completed independently by the patients before surgery and after one year postoperatively (8).

The global health status was calculated following established guideline, where higher scores were associated with an increased HRQoL (9, 10). Voiding patterns and continence statuses were evaluated using the same questionnaire. Continence rates and time intervals between clear intermittent catheterizations (CICs) at last follow-up were recorded. Urinary continence was defined as absence of urine leakage between self-catheterizations (11). The neobladder capacity and post-void residual urine volume were also evaluated. CICs was recommended for patients with a post-void residual of >100mL. Each complication that occurred was classified as early (<3 months) or late (>3 months after surgery).

### Statistical Analysis

Data were expressed as mean±standard deviations (SD) or percentage (%). The Student T test and Mann Whitney U test were used to determine statistical significance. In all the analyses, a p value of statistical analysis was performed with SPPS v16.0 (SPPS, Chicago, IL, USA).

## RESULTS

Our study enrolled 22 patients, who underwent RC with standard pelvic lymph node dissection. Clinicopathological features and perioperative outcomes are summarized in Table-1.

**Table 1:** Clinicopathological features and perioperative outcomes with diversion-related complications within first three months and during follow-up after surgery with their management.

Mean (SD) / Percentage (%)			
Demographic & pathological characteristics		Patients (n=22)	
Age (years)		61.2 ± 8.1	
BMI, kg/m^2^, mean		27.2 ± 2.6	
Male/Female, n (%)		18 ± 82 / 4 ± 18	
Hospital stay (days)		19 ± 3.4	
Maximum neobladder capacity (mL)		453 ± 61.4	
Postvoid urinary volume (PVR), (mL)		83.8 ± 35.5	
**Reservoir related complications, n (%)**		**≤ 3 months**	**> 3 months**
VUR, n (%)		3 (13.6)	2 (9)
Gr I -II		3	2
Gr III -IV		0	0
**Hydronephrosis, n (%)**		5 (22.7)	3 (13.6)
Unilateral		0	1
Bilateral		5	2
Pyelonephritis, n (%)		2 (9)	2 (9)
i.v antibiotics only		1	0
oral antibiotics only		1	2
Anastomotic leakage rate, n (%)		1 (4.5)	0
Anastomotic stricture rate, n (%)		0	0
Metabolic acidosis, n (%)		4 (18.1)*	1 (4.5)
Complications treatment, n (%)		11 (50)*	5 (22.7)
Intermitent catheterization (CICs)		4	2
Sodium bicarbonate (i.v/oral)		3/1	0/1
Antibiotics (i.v/oral)		1/1	0/2
JJ stent placement		1	0

* statistically significant difference (p<0.05)

**Figure 3:**
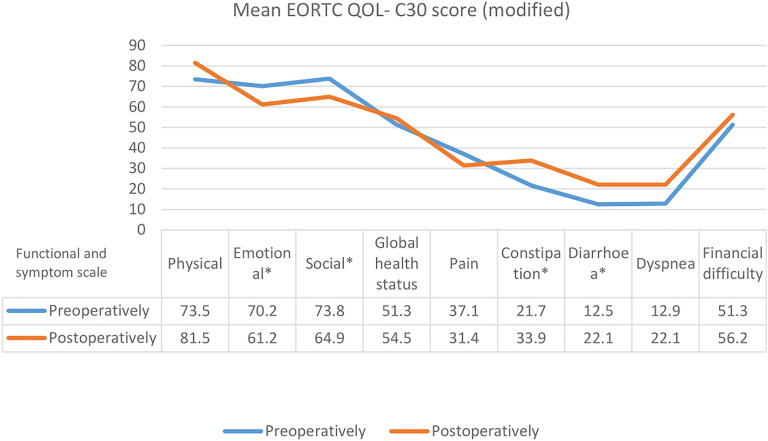
EORTC QLQ-C30 scores preoperatively and at the end of the 12-month follow-up period. Asterisk (*) denotes statistically significant difference between samples (p <0.05).

All patients had transition cellular carcinoma and the tumor stage ranged from T2 to T3 N0-3/M0. The pathological stage was pT3 in 5 and pT2 in 17 patients, where 15 patients had high grade (G3) disease. Neoadjuvant chemotherapy was performed in 8 patients (36.3%). The average age of the patients was 61.2±8.1 years (45 to 74 years). The total operative duration was 240±33.6 min and the estimated blood loss 400±210mL. Patients follow-ups were 13 to 48 months (mean 29.4±10.3) and distribution of reservoir-related complications and their management categorized by the time of occurrence. Within the first three months post surgery, hydronephrosis was observed in five patients (18%), while VUR occurred in three patients (13.6%) respectively. No UIS was detected as a cause of hydronephrosis within the first three months or afterwards. However, the anastomotic leakage rate was 4.5% during the early postoperative period. Four patients (18.1%) developed metabolic acidosis, three months post-operatively and management consisted of immediate intravenous sodium bicarbonate administration with oral solution maintenance for four weeks (grade II Clavien Classification of Surgical complications). During the follow-up, two patients with persistent VUR had improved after CICs (grade I Clavien). In addition, all patients with hydronephrosis had improved with or without treatment (grade I-II Clavien). Moreover, one patient with anastomotic leakage was treated with retrograde bilateral JJ stent insertion, antibiotics and CICs (grade IIIa Clavien) (Table-2). No local tumor recurrence took place. Serum creatinine was less than 1.4mg/dL pre-operatively in the majority of patients and remained within normal ranges. There was no statistically significant difference in change in estimated glomerular filtration rate until last follow-up (74.2 vs. 66.7mL/min/1.73m2, p=0.32). During the last follow-up visit, complete daytime continence was reported in 18 patients (81.8%), whereas complete nighttime continence was achieved in 16 patients (72.7%). Incontinent patients had to catheterize themselves every 4-8hrs. With regards to quality of life, patient-reported HRQoL decreased significantly post-operatively, while symptoms score aspects were maintained (Figure-3).

**Table 2:** Clavien - Dindo classification of diversion - related complications and its incidence during early (≤3 months) and late (>3 months) postoperative follow-up period.

Mean / Percentage (%)		
Postoperative complications (Clavien - Dindo classification)	Patients (n=22)	
	Early	Late
Grade I	7 (31.8)	5 (22.7)
Grade II	7 (31.8)*	3 (13.6)
Grade III (IIIa/IIIb)	1 (4.5)	0
Grade IV (IVa/IVb)	0	0

* statistically significant difference (p<0.05)

## DISCUSSION

The first modification of the original Hautmann technique was published by Lippert & Theodorescu in the late nineties (1). Several years later, Hollowell et al. (2) claimed that the technique of Hautmann neobladder with chimney modification is safe and feasible and has favorable surgical outcomes compared to other techniques. The common issue for these reports is the type of uretero-ileal anastomosis performed in a direct, end-to-side manner (Bricker). This approach may be associated with a relatively high rate of UIS, ranging from 6-20% (12, 13). The findings we reported here support this statement, since the main reason for developing the new technique in our institution was a high incidence of UIS after neobladder construction with Bricker uretero-ileal anastomosis. Nevertheless, our recent study (6) reported significantly lower anastomotic stricture and anastomotic leakage rates using modified Wallace technique in uretero-ileal anastomosis, which could be important issues in minimizing both short- and long-term postoperative complications. Moreover, Hautmann et al. (14) claimed that freely refluxing Wallace anastomosis to the afferent limb of the orthotopic reservoir has the lowest non-tumor related anastomotic stricture rate (5.4% comparing to 16.3% using Bricker technique). Furthermore, the same author emphasized the importance of the anastomotic technique instead of chimney length in preventing strictures and reflux.

It can be deduced that Wallace anastomosis on modified chimney has paramount importance in reducing UIS rate. Indeed, in our study group, none of the patients developed UIS, after a median 29 months follow-up, with anastomotic leakage rate of only 4.5%. Overall, the incidence of early and late postoperative, reservoir-related complications, were 68.1% and 36.3%, respectively, but neither UIS nor anastomotic leakage were detected three months postoperatively. This is an important issue and supports our technique on stricture formation and leakage occurrence. This is possibly due to Wallace uretero-ileal anastomosis, short chimney and long ureteral spatulation, with sparing as much ureteral length as possible. Indeed, Shigemura et al. (15) claims that the advantages of Wallace anastomosis over the Bricker technique are depicted in conjoined ureteral plate and larger diameter for uretero-ileal anastomosis, with less tension on the ureteral walls that ultimately resulted in the absence of UIS and anastomotic leakage. Moreover, technical simplicity, shorter operative time and easiness in performing retrograde pyelography during the follow-up period, were all advantages of the anastomotic technique in our study group. Bianchi G et al. (16) claimed that Wallace anastomosis in modified Studer neobladder reduces the risk of VUR probably due to straight, long conductive ureters with no ureteral kinking. Nevertheless, a shorter chimney may also play a role in reflux prevention, due to the fact that a shorter afferent limb allows the use of longer segments of the lower ureters that participate in reflux prevention (17). All these findings together bolster the assertion that meticulous ureteral handling, along with fine suturing technique of Wallace uretero-ileal anastomosis and shorter intestinal chimney, are essential to minimize the risk of postoperative strictures, urinary leakage and VUR. However, studies presenting different techniques of uretero-ileal anastomosis and orthotopic reservoir construction, showed comparable results in regard to early and late postoperative complications rate (18, 19).

The ideal form of urinary diversion would approximate normal bladder function and provide continent, non-refluxing, low-pressure storage of sterile urine, and allow complete and convenient emptying. We presented here the technique relatively easy to perform, which creates a reliable uretero-intestinal anastomosis without tension. In addition, 49-52cm of detubularized ileum used to create pouch reservoir guarantees proper reservoir capacity of 400-500mL at 3-6 months, provided that no significant reflux to the upper tract occurred. Incidence of VUR in our study was 13.6% after the first three months and 9% during follow-up time, with grade I-II presented in 4 out of 5 cases, which definitely accelerated proper capacity formation.

Nevertheless, this volume reduces the incidence of metabolic disorders, e.g. acid-base and electrolyte imbalances, since 60cm of ileal segment usually leads to a large absorbing area of the neobladder mucosa and hyperchloremic metabolic acidosis (8, 9). The rate of metabolic acidosis was 18.1% during first three months and 4.5% afterwards, with mild symptoms and oral alkalizing therapy. Our results are encouraging compared to several studies where even shorter ileal segments for pouch construction (40cm) were associated with a higher incidence of metabolic acidosis of up to 58% (20, 21). Therefore, high volume, low-pressure orthotopic bladder substitute, together with short chimney and long, conductive ureters is essential point in upper tract preservation and prevention of metabolic acidosis. Additionally, it doesn’t seem that the site of the orthotopic neobladder outlet (non-hole vs. button-hole technique) is associated with a significant increase in the complication rate (22).

Patients with neobladder reservoir who should depend on CIC immediately after RC can experience similar negative impacts on their quality of life. In our study, two patients (9%) still required CICs three months after surgery, with complete daytime and nighttime continence achieved in 81.6% and 72.7% respectively. These results were comparable to those reported in other studies (23, 24).

The limitations of our study are small sample size and a short follow-up period. Additionally, we compared our results mostly with a small cohort of studies, describing Hautmann neobladder with modified chimney technique, thus additional studies may be necessary to strengthen our results. Despite that, we found acceptable rate of uretero-enteric strictures, VUR and anastomotic leakage, lower than those in the conventional technique.

## CONCLUSIONS

Wallace direct uretero-ileal anastomosis when combined with short, afferent isoperistaltic chimney is an effective way to minimize potential UIS, anastomotic leakage and incidence of VUR. This is a simple surgical technique in Hautmann neobladder with chimney modification.

## LIST OF ABBREVIATIONS

UIS: uretero-ileal anastomotic stricture
VUR: vesicoureteral reflux
HRQoL: Health related quality of life
CIC: Clean intermittent catheterization
RC: radical cystectomy
Hr: hours
